# Microbial polyhydroxyalkanote synthesis repression protein PhaR as an affinity tag for recombinant protein purification

**DOI:** 10.1186/1475-2859-9-28

**Published:** 2010-05-10

**Authors:** Shuang Zhang, Zhi Hui Wang, Guo Qiang Chen

**Affiliations:** 1 Multidisciplinary Research Center, Shantou University, Shantou 515063, Guangdong, China; 2 Department of Biological Sciences and Biotechnology, School of Life Science, Tsinghua University, Beijing 100084, China

## Abstract

**Background:**

PhaR which is a repressor protein for microbial polyhydroxyalkanoates (PHA) biosynthesis, is able to attach to bacterial PHA granules *in vivo*, was developed as an affinity tag for *in vitro *protein purification. Fusion of PhaR-tagged self-cleavable Ssp DnaB intein to the N-terminus of a target protein allowed protein purification with a pH and temperature shift. During the process, the target protein was released to the supernatant while PhaR-tagged intein was still immobilized on the PHA nanoparticles which were then separated by centrifugation.

**Results:**

Fusion protein PhaR-intein-target protein was expressed in recombinant *Escherichia coli*. The cell lysates after sonication and centrifugation were collected and then incubated with PHA nanoparticles to allow sufficient absorption onto the PHA nanoparticles. After several washing processes, self-cleavage of intein was triggered by pH and temperature shift. As a result, the target protein was released from the particles and purified after centrifugation. As target proteins, enhanced green fluorescent protein (EGFP), maltose binding protein (MBP) and β-galactosidase (lacZ), were successfully purified using the PhaR based protein purification method.

**Conclusion:**

The successful purification of EGFP, MBP and LacZ indicated the feasibility of this PhaR based *in vitro *purification system. Moreover, the elements used in this system can be easily obtained and prepared by users themselves, so they can set up a simple protein purification strategy by themselves according to the PhaR method, which provides another choice instead of expensive commercial protein purification systems.

## Background

Fusion protein technology has been enthusiastically developed since the advent of genetic engineering. It is a convenient way to simplify the purification scheme by adding a molecular handle to a recombinant protein [1,2]. A large amount of protein purification tags are available now [3], such as polyhistidine (Poly-His), maltose-binding protein (MBP) and glutathione S-transferase (GST) [4-6]. However, the cost of affinity resins and other required reagents are the major obstacles that hinder these tags to be developed into large-scale protein production.

Polyhydroxyalkanoates (PHA) are a family of hydrophobic biopolyesters produced by many bacteria [7]. Several types of proteins were found to attach on the surface of *in vivo *PHA granules, such as PHA synthase (PhaC), PHA depolymerase (PhaZ), granule associated protein (PhaP) and repressor protein (PhaR) [8-11]. Some studies showed that PhaR in the crude lysates of recombinant *E. coli *was able to rebind to poly [(*R*)-3-hydroxybutyrate] (PHB) granules [12-14]. PhaR has two separate domains, including a DNA sequence binding domain and a PHB granule binding domain [13]. Previous study proved that PhaR was able to tightly attach to both artificial amorphous and crystalline PHB granules *in vitro *[13]. In addition, PhaR was reported to bind to the surface of polyethylene, polystyrene and poly-lactic acid, which shows the binding is non-specific, mainly by hydrophobic interaction [14].

Intein-based controllable cleavages have been adopted in increasing number of applications including the IMPACT method for single-step purification of recombinant proteins [15-17], the expressed protein ligation (EPL) method for protein ligation [18,19], and the cyclization of recombinant proteins or peptides [20]. In a more recent study [21], Ssp DnaB mini-intein was shown as a promising tool for production of peptides of pharmaceutical interest [22].

Banki et al invented the intein-mediated protein purification method using the in vivo PHB matrix association phenomenon [8]. Since self-cleavage of intein is simply induced by pH and temperature shift, it is becoming important for various protein studies.

In our previous study, PhaP, the major binding protein on *in vivo *PHA granules, was developed as an affinity tag used for recombinant protein purification [23]. In this paper, PhaR replaced PhaP for the purification system, PhaR-intein tagged proteins expressed in recombinant *E. coli *were released together with all other *E. coli *proteins *via *a bacteria lysis process, they were bound to the surface of PHA nanoparticles prepared *in vitro*. Similar to the PhaP based system, PhaR based method was successfully used to purify green fluorescent protein (EGFP), maltose-binding protein (MBP) and β-galactosidase (LacZ) *in vitro*.

## Materials and methods

### Construction of plasmids

Plasmid pPIEGFP [23] available in our laboratory was modified to generate corresponding proteins encoding tripartite fusions of PhaR, Ssp DnaB mini-intein and target proteins. Primers R1 (5'-GGA**AGATCT**ATGGCCACGACCAA-3') and R2 (5'-CCC**AAGCTT**TTACTTCTTGTCCG-3') were used to amplify *phaR *gene (GenBank: NC 008313) from *Ralstonia eutropha *H16 genome. Then *Hind*III and *Bgl*II digested *phaR *segment was inserted into the corresponding site of IE segment amplified from pPIEGFP to generate pRIEGFP using following primers: IE1 (5'-CCC**AAGCTT**AACAACGGTAACAACGGTCTC-3') and IE2 (5'-GGA**AGATCT**ATGTATATCTCCTTCTTAAA-3'). To simplify plasmid constructions, *BamH*I and *Xma*I sites were added to the terminals of RI segment PCR amplified from pRIEGFP using primers RI1 (5'-CGC**GGATCC**GGCTGCTAACAAAG-3') and RI2 (5'-CCC**CCCGGG**GGAAGAGCCCTCGAGGAATT-3'). The *lacZ *gene encoding β-galactosidase was PCR amplified from *E. coli *S17-1 (ATCC 47055) genome employing primers IZ1 (5'-CGC**GGATCC**TTATTTTTGACACCAGACCA-3') and IZ2 (5'-CCC**CCCGGG**ACCATGATTACGGATTCACT-3'). Similarly, MBP gene was obtained from pMAL-c2x (NEB) using primers M1 (5'-CGC**GGATCC**CAACATGAAAATCGAAGAAGGTAAACTGG-3') and M2 (5'-CCC**CCCGGG**TTATCGAGCTCGAATTAGTCTGCG-3'). The *BamH*I and *Xma*I digested MBP and *lacZ *genes were then inserted into corresponding sites of RI segment to generate plasmids pRI-MBP and pRI-LacZ, respectively.

### Protein expression and collection of crude cell lysates

Plasmids pRIEGFP, pRI-LacZ and pRI-MBP were transformed into *E. coli *BL21 (DE3) for expression of PhaR-intein tagged proteins, respectively. All recombinant strains were cultivated in 1000 ml shake flasks containing 200 ml Luria Bertani medium (1% w/v Bacto tryptone, 0.5% yeast extract and 1% NaCl) supplemented with 100 μg ml^-1 ^ampicillin in a rotary incubator at 200 rpm. *E. coli *BL21 (DE3) transformants carrying corresponding plasmids were diluted 100: 1 from overnight cultures and grown at 37°C. At OD_600 _= 0.5, 1 mM isopropyl β-D-thiogalactopyranoside (IPTG) was added to induce the expression of fusion genes at 15°C overnight. Cells were harvested by centrifugation at 11000 rpm for 2 min and re-suspended in a binding buffer (20 ml 20 mM Tris-Cl pH 8.5) followed by sonication (Ultrasonic crasher, Scientz-II D, Ningbo, China) for 10 min at 50% output. After centrifugation at 11000 rpm and 4°C for 30 min, the clarified soluble protein fraction was collected and stored at -20°C.

### Preparation of PHA nanoparticles

**Figure 1:**
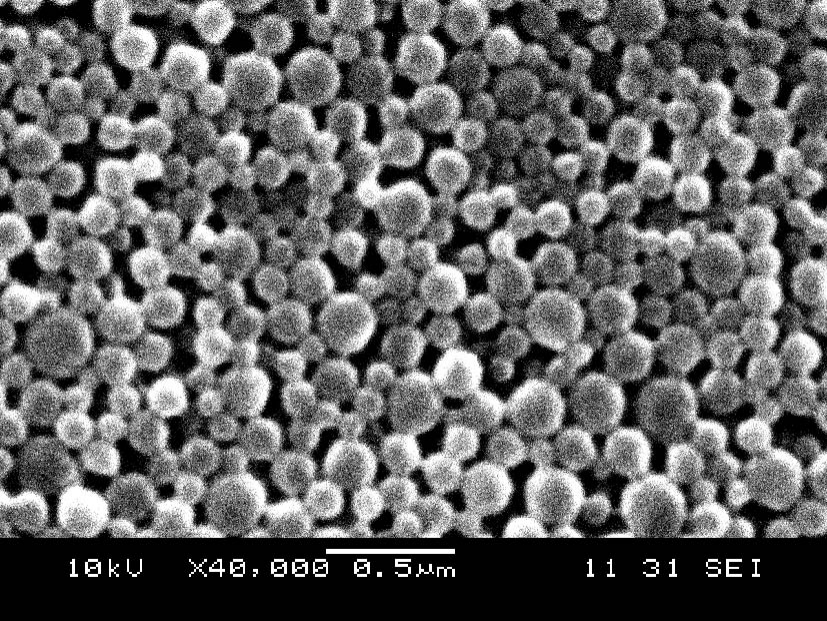
**SEM image of PHBHHx nanoparticles**.

PHA nanoparticles were prepared by the oil-in-water emulsion method described previously [23]. In detail, 5% w/v PHBHHx (copolymer of 88 mol% 3-hydroxybutyrate and 12 mol% 3-hydroxyhexanoate, a member of PHA polymer family) powders were dissolved in chloroform. Subsequently, 10 mM sodium oleate aqueous solution was added to the chloroform solution in a volume ratio of 1: 20. The mixed solution was emulsified by sonication for a total 3 min at 50% output. The 3 minutes sonication was carried out using a process of 1 second sonication followed by 3 seconds pause to avoid overheating. The chloroform was removed from the emulsion by a rotary evaporator (RE-52AA, Shanghai, China) under vacuum conditions. PHBHHx nanoparticles were harvested by centrifugation at 11000 rpm for 10 min and washed twice with an equal volume of distilled water. Then nanoparticles were lyophilized for 24 h in a freeze dryer (ModulyoD, Thermo, USA). The morphological analysis was performed using scanning electron microscopy (SEM, JSM-6360LA, Japan) and the approximate particle size was evaluated (Fig. 1).

### Self-cleavage of intein and the release of target proteins

**Figure 2:**
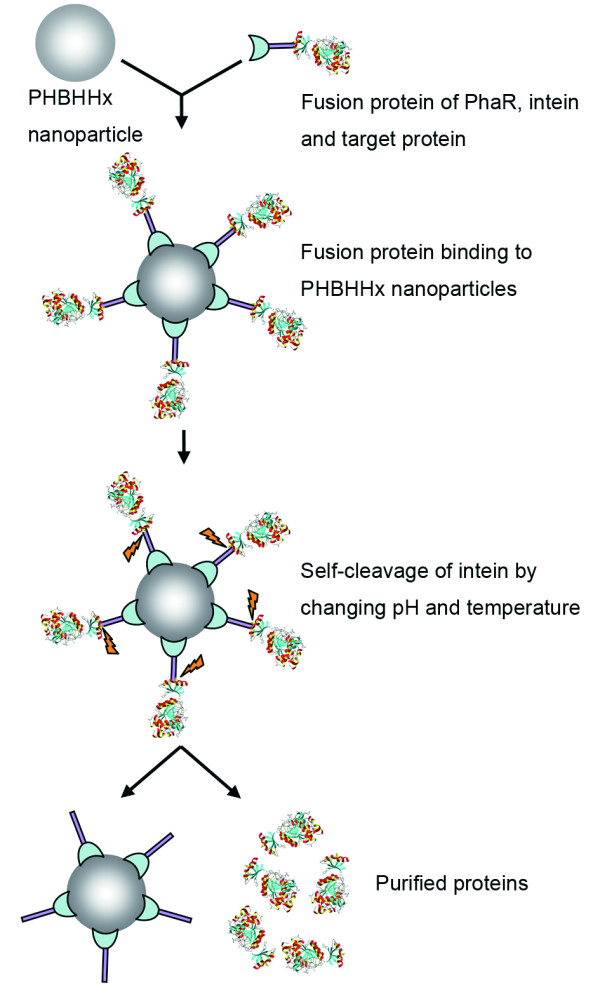
**A schematic illustration for PhaR based protein purification system**. Fusion proteins of PhaR, intein and a target protein were expressed in *E. coli *BL21 (DE3), the cellular supernatant obtained after sonication and centrifugation contained all crude proteins that were subjected to incubation with PHBHHx nanoparticles. After the overnight incubation at 4°C, the pellets were harvested and washed to remove unbound proteins. Subsequently, cleavage buffer was used to trigger self-cleavage of intein at 25°C. The target protein was released into the supernatant and separated from the pellets by centrifugation after cleavage incubation.

The schematic illustration for nanoparticle based protein purification is described below (Fig. 2): 4 ml Crude cell lysates were incubated with 100 mg of PHBHHx nanoparticles in binding buffer (20 mM Tris-Cl, pH 8.5) at 4°C overnight to allow sufficient attachment. PHBHHx nanoparticles coated with fusion proteins were collected by centrifugation at 11000 rpm and 4°C for 10 min and washed twice with 10 ml binding buffer, then re-suspended in 1 ml cleavage buffer (20 mM Tris-Cl, pH 7.0) at 25°C. In the cleavage process, a spot of pellets were taken, washed and resolved by SDS-PAGE every other hour to identify the completion of cleavage reaction. Finally, the target proteins were completely released to supernatant, and were separated with particles by centrifugation at 15000 rpm and 4°C for 5 min. Samples in each step were collected, dissolved in SDS-PAGE sample loading buffer (Beyotime, Jiangsu, China), followed by being boiled for 5 min. The supernatant was assayed by 12.5% SDS-PAGE after centrifugation.

### Assays of protein activities and yields

Protein concentration was measured as described in Bradford's method [24] with a protein assay kit (Tiangen, Beijing, China). The fluorescence intensity of purified EGFP was visually inspected under a fluorescence microscope. MBP was considered to be active if it can bind to maltose resin. β-Galactosidase activity assay based on its reaction with *o*-Nitrophenyl-β-D-galactopyranoside (ONPG) was measured by β-galactosidase assay kit (GMS60003.3, GENMED Scientifics Inc. USA). One unit of β-galactosidase hydrolyzes 1.0 μmol ONPG to *o*-nitrophenol and D-galactose per minute at 37°C.

## Results

### Purification of three types of proteins and SDS-PAGE analysis

**Figure 3:**
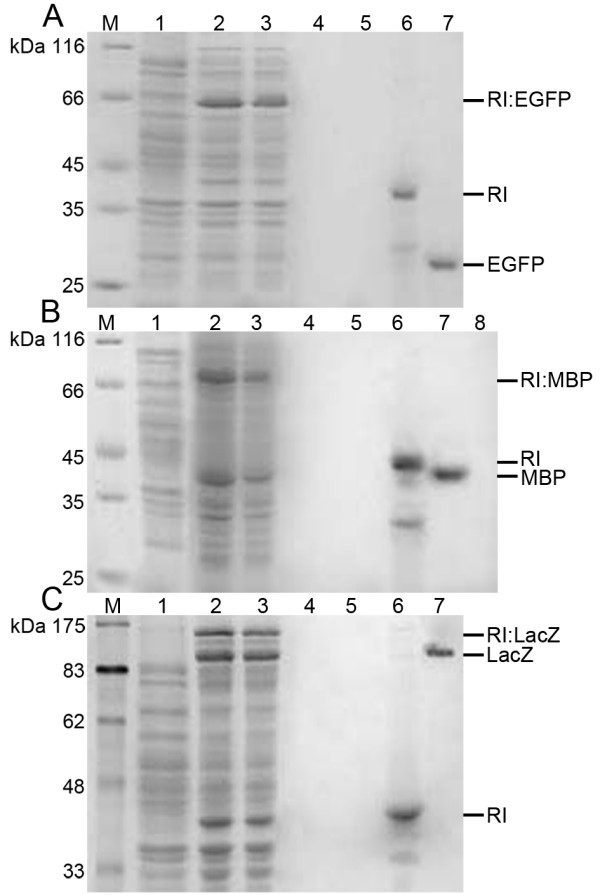
**SDS-PAGE analysis of three protein samples subjected to the purification process**. A: enhanced green fluorescent protein (EGFP); B: maltose binding protein (MBP) and C: β-galactosidase (LacZ). RI: PhaR-intein segment. Lane M, protein molecular weight standards; Lane 1: Pre-induced whole cell lysate; Lane 2: Soluble fractions (crude proteins) of induced cell lysate; Lane 3: Proteins absorbed on nanoparticles; Lanes 4 and 5: First and second eluates; Lane 6: Pellets after self-cleavage of intein; Lane 7: Supernatant after self-cleavage of intein; Lane 8: Supernatant after co-incubation with maltose resin.

Three proteins were used to study the feasibility of the PhaR based system, including enhanced green fluorescent protein (EGFP), maltose binding protein (MBP) and β-galactosidase (LacZ) (Fig. 3). Three distinct bands of fusion proteins PhaR-intein-EGFP (RI:EGFP) (Fig. 3A, lane 2), PhaR-intein-MBP (RI:MBP) (Fig. 3B, lane 2) and PhaR-intein-LacZ (RI:LacZ) (Fig. 3C, lane 2) in soluble cell lysates were observed compared with pre-induced whole cell lysate (Fig. 3, lane 1). After co-incubation of soluble cell lysates and PHBHHx nanoparticles, proteins attached on PHBHHx nanoparticles were shown in lane 3. The repeated washing process using washing buffer excessively diluted the released proteins from the PHBHHx particles, this was the reason that no band was observed in lanes 4 and 5, the small amount of the eluate used for SDS-PAGE was also one of the reasons. After the induced self-cleavage of intein, the PhaR-intein (RI) segment was still immobilized on the particle surface (Fig. 3, lane 6), while the purified target proteins were released to the supernatants (Fig. 3, lane 7).

### Assays of purified protein activity

The purified EGFP emitted green fluorescence in natural light, and the fluorescence became stronger when excited by light of 460-500 nm, which implied that EGFP was still active after the PhaR based purification process. Activity of the purified MBP was confirmed by its affinity to maltose resin. After co-incubation of the purified MBP and the maltose resin, no MBP was found in the supernatant (Fig. 3B, lane 8). As shown in table 1, the activity of LacZ in induced cells was greatly enhanced to 167 units compared with only 7 units in pre-induced cells, indicating the expression of active LacZ fusion. And the purified LacZ had a much higher activity (487 units) than its fusion (167 units) in cell lysate. The average yields based on nanoparticle weight and wet bacterial weight were calculated (Table 1). The efficiencies of the PhaR based system was found to be similar to the PhaP based one reported by our lab previously [23]. However, PhaR system seemed to be more effective for protein purification compared with that of the PhaP based in vivo system developed by Banki et al [8]. For example, the PhaR system developed in this study produced 4.01 mg MBP/g wet weight of bacteria compared with only 1.34 mg from Banki's system [8].

**Table 1:** Effect of PhaR based protein purification method for purifying EGFP, MBP and LacZ, respectively

	**Yield of purified protein**		**LacZ activity (Units/mg ^a^)**		
		
**Target proteins**	**mg/g nanoparticles**	**mg/g wet weight of bacteria**	**Pre-induced whole cell lysate**	**Induced whole cell lysate**	**Purified LacZ**
	
EGFP	9.14 ± 1.2	4.39 ± 0.5			
MBP	8.17 ± 1.06	4.01 ± 0.3			
LacZ	6.55 ± 0.9	3.16 ± 0.3	7.22 ± 0.9	167 ± 7.5	486.77 ± 15

^a ^One unit of β-galactosidase activity hydrolyzes 1.0 μmol ONPG to *o*-nitrophenol and D-galactose per minute at 37°C.

## Discussion

In this study, a strong and controllable T7lac promoter was employed to ensure high-level expression of three fusion proteins, respectively. In order to avoid formation of protein inclusion bodies, fusion protein expressions were undertaken at temperature as low as 15°C. In addition, a flexible peptide linker was inserted between PhaR and intein to ensure their correct folding. As a result, all three soluble fusion proteins were successfully expressed (Fig. 3, lane 2). However, undesired premature self-cleavage still occurred (Fig. 3, lane 2), the truncated PhaR-intein segment would compete with intact tripartite fusion for binding to nanoparticles. To tackle this problem, a sulfhydryl group induced intein was used to replace pH and temperature induced intein. However, no soluble fusion proteins were recovered (data not shown).

In addition to PhaR-intein segment, small amount of cellular proteins could also bind to the PHA (PHBHHx) nanoparticles even though PhaR tagged fusion protein was the dominant protein on the nanoparticle surface (Fig. 3, lane 3). The binding of other cellular proteins on PHA nanoparticles reduced the loading capacity of target proteins, leading to reduced efficiency of target protein purification. Nevertheless, increased yield of target protein purification was still achieved compared with that of the Banki's in vivo system. The separation of fusion protein production and PHA nanoparticle preparation allows the use of large amount of PHA nanoparticles to be attached by fusion proteins of PhaR-intein-target proteins, thus could improve the recovered efficiency of the target proteins. On one hand, both protein expression and PHA nanoparticle preparation can be conveniently achieved. In contrast, the in vivo PHA granule accumulation is indeed a more complicated process, the extraction of native PHA granules with target proteins attached on their surface is time-consuming and laborious, resulting in significant loss of target proteins. On the other hand, PHA nanoparticles can be prepared in sufficient quantity to allow all PhaR-intein-tagged proteins to absorb (Fig. 1). Proteins purified by the in vitro method were reasonably pure, no contaminant protein was found in purified protein solution (Fig. 3, lane 7). Only several steps of mild washing and centrifugation processes were required throughout this PhaR based purification course, other treatment conditions were so mild that a high activity of target protein can be maintained after the whole purification process as demonstrated by the high LacZ activity (Table 1) and binding ability of MBP (Fig. 3B, lane 8). Simultaneously, strong green fluorescence of purified EGFP also indicated its activity.

The *in vivo *PHA matrix and PhaP based protein purification system invented by Banki et al was cost-effective and suitable for large-scale protein production [8]. The method is more suitable for purifying prokaryotic proteins as accumulation of PHA granules in eukaryotic microorganisms are rare. This problem can be easily addressed by separating protein expression and PHA nanoparticle preparation processes, wherein PhaR tagged proteins can be expressed freely in either prokaryotic or eukaryotic organisms, allowing the PhaR based system for the purification of eukaryotic proteins. This *in vitro *method separating protein expression and PHA nanoparticles preparation widens the applications of Banki's method.

Besides PHA, PhaR can also attach to polyethylene, polystyrene and poly(lactic acid) et al [14,23]. These hydrophobic polymers can also be used to prepare nanoparticles to be used in the PhaR system. In this study we chose PHBHHx as affinity matrix only because it was available in large quantities in our laboratory. And it is easier and much more cost-effective to prepare nanoparticles than conventional chromatographic column. Sizes and shapes of nanoparticles may not be important although smaller size will give larger surface area. Importantly, the nanoparticles used in this purification system can be easily prepared by all users themselves. Ideally, researchers can set up a simple protein purification strategy by themselves according to the PhaR method, it is not necessary to buy an expensive protein purification system, when they just need small amount of pure proteins. Certainly, the amount of purified protein depends on how many nanoparticles are used.

Intein mediated self-cleavage system has been developed as a powerful tool for protein purification, protein ligation, and peptide amidation [16,19,25-27]. The intein-based methods sometimes involve minimal risk of non-specific cleavage at unintended locations within the target proteins, yet intein is still effective compared with some protease-based methods [28-31]. Completely controllable cleavage of intein and target proteins is still a challenge, in which the cleavage reaction is induced only when needed, i.e., after the intein-target protein complex has been expressed and purified successfully.

The data presented here have proven the feasibility of this PhaR based protein purification system. However, some short-comings still need to be improved. Firstly, some nanoparticles often aggregated during the centrifugation processes, it was difficult to re-suspend them, this reduced the final yield of target protein. Secondly, the complete cleavage of intein took as long as 24 h, which may result in reduction of target protein activity. An intein cleavage with a short cleavage time is desirable.

## Conclusions

The *in vitro *purification system based on intein and PhaR as the affinity tag able to attach to the surface of PHA nanoparticles (in this case, PHBHHx) was used successfully for purifying three proteins including EGFP, MBP and LacZ. Comparable to several other PHA bind proteins including PhaP, PhaZ and possibly PhaC, PhaR, as an affinity tag, is at least as good as others for pure recombinant protein production. Moreover, PhaR (20 kDa) is smaller than PhaP (21 kDa), PhaZ (45 kDa) and PhaC (65 kDa) in *Ralstonia eutropha *H16, therefore, its expression and accumulation should be more effective and richer compared with others.

## Competing interests

The authors declare that they have no competing interests.

## Authors' contributions

ZHW designed the study and participated in the whole process of the experiments, SZ performed the experiments and drafted the manuscript, GQC supervised the study. All authors read and approved the final manuscript.
